# Clinical and Anatomopathological Evaluation of BALB/c Murine Models Infected with Isolates of Seven Pathogenic *Sporothrix* Species

**DOI:** 10.3390/pathogens10121647

**Published:** 2021-12-20

**Authors:** Danielly Corrêa-Moreira, Rodrigo C. Menezes, Orazio Romeo, Cintia M. Borba, Manoel M. E. Oliveira

**Affiliations:** 1 Department of Education, Clinical Research of Infectious Diseases, Evandro Chagas National Institute of Infectious Diseases, Rio de Janeiro 21040-360, Brazil; 2 Laboratory of Taxonomy, Biochemistry and Bioprospecting of Fungi, Oswaldo Cruz Institute, Rio de Janeiro 21040-360, Brazil; cintiaborba@terra.com.br (C.M.B.); manoel.marques@ioc.fiocruz.br (M.M.E.O.); 3 Laboratory of Clinical Research in Dermatozoonoses in Domestic Animals, Evandro Chagas National Institute of Infectious Diseases, Rio de Janeiro 21040-360, Brazil; rodrigo.menezes@ini.fiocruz.br; 4 Department of Chemical, Biological, Pharmaceutical and Environmental Sciences, University of Messina, 98100 Messina, Italy; orazio.romeo@unime.it

**Keywords:** *Sporothrix*, sporotrichosis, experimental mice model, virulence, pathogenic clade, environmental clade, host-parasite relationship

## Abstract

Background: Sporotrichosis is a subcutaneous mycosis with worldwide distribution and caused by seven pathogenic species of *Sporothrix* genus: *S. schenckii* sensu stricto, *S. brasiliensis*, *S. globosa* and *S. luriei* (clinical clade), and the species *S. mexicana*, *S. pallida* and *S. chilensis* (environmental clade). Isolates of the same species of *Sporothrix* may have different pathogenicities; however, few isolates of this fungus have been studied. Thus, the aim of this work was to analyze the clinical and anatomopathological changes in immunocompetent and immunosuppressed BALB/c mice infected with clinical and environmental isolates of seven different species of *Sporothrix*, from both clades. One human clinical isolate of *S. schenckii* sensu stricto, *S. brasiliensis*, *S. globosa*, *S. luriei*, *S. mexicana* and *S. chilensis* species and one environmental isolate of *S. pallida* were inoculated subcutaneously in immunocompetent mice and the same isolates of *S. brasiliensis* and *S.* *schenckii sensu stricto* were inoculated in immunossupressed mice. Clinical manifestations as external lesions, apathy, and alopecia were observed. At 21, 35, and 49 days after fungal inoculation, four mice from each group were weighed, euthanized and necropsied for evaluation of splenic index, recovery of fungal cells, macroscopic and histopathological analysis of livers, lungs, kidneys, and hearts. The survival assessment was observed for 50 days following inoculation. Our results demonstrated that, clinical *S. schenckii* isolate, followed by clinical *S. mexicana*, and environmental *S. pallida* isolates, the last two, species grouped in the environmental clade, were capable of inducing greater anatomopathological changes in mice, which was reflected in the severity of the clinical signs of these animals. Thus, we reinforce the hypothesis that the pathogenicity of *Sporothrix* is not only related to the species of this fungus, but also shows variation between different isolates of the same species.

## 1. Introduction

The *Sporothrix* genus is composed of about 60 species distributed worldwide in the tropical and subtropical regions, being commonly saprophytes. Until 2006, *S. schenckii* was considered the only pathogenic species; however, phylogenetic studies have demonstrated genetic variability within them [1]. From then on, *S. schenckii* was considered a species complex, initially composed of *S. schenckii sensu stricto*, *S. brasiliensis*, *S. globosa*, *S. luriei*, *S. mexicana*, and *S. pallida* species, all currently considered pathogenic [2,3,4,5,6,7,8]. Additionally, multigenic analyses of isolates initially characterized as *S. pallida* from Chile revealed the presence of a new species, *S. chilensis* [9], also described in Brazil [10], as pathogenic for humans.

Sporotrichosis is a worldwide subcutaneous mycosis endemic in Latin America [11] and hyperendemic in the metropolitan region of Rio de Janeiro state, Brazil, where the largest number of cases in the world occurred (over 5000 cases in humans and 4000 cases in animals since 1998). In this region, the main form of transmission is zoonotic rather than the classical transmission (through contaminated plants, wood, or soil), and the main species involved in human and animal cases is *S. brasiliensis* [12,13]. Although this last species was restricted to Brazil, it is currently expanding to Argentina and Paraguay [14]. The species *S. globosa* is the most common in human cases in Asia and America, and in Europe the first autochthonous was reported in Portugal [15,16,17]. The species *S. schenckii sensu stricto* predominate in humans in Australia, South Africa, western South and Central America, and North America and is the only species described in cats in Malaysia [18]. The species *S. mexicana* is most commonly isolated from environment and rarely reported in human clinical cases in Brazil [19] and Portugal [5]. The species *S. lurei* is a rare fungus of clinical origin isolated from human patient in South Africa [3] and from a dog with sporotrichosis in Brazil [20]. Although rarely associated with clinical cases, *S. pallida* and *S. chilensis* were recently reported respectively in a cat in Australia [21] and in a human Brazil [10].

Since the 1940s, mammalian models (predominantly mice) infected by different routes (subcutaneous, intravenous, intraperitoneal) and with different forms (mycelial or yeasts) have been used to evaluate differences in pathogenicity among *Sporothrix* isolates [22]. We believe that the studies using experimental models are of paramount importance so that we can understand the different biological behaviors of species that infect mammalian hosts and hereafter, the response of these hosts to the infection with *Sporothrix* species. Thus, our aim was to analyze the clinical and anatomopathological changes in immunocompetent and immunosuppressed BALB/c mice infected with clinical and environmental isolates of different species belonging the pathogenic and environmental clades of *Sporothrix* [23].

## 2. Results

### 2.1. In Vivo Assessment of the Sporothrix Species

Immunocompetent mice infected (ICI) with the seven *Sporothrix* species and control group (ICC) gained weight throughout the experimental period, with no statistical differences among the groups (Table 1).

**Figure 1:**
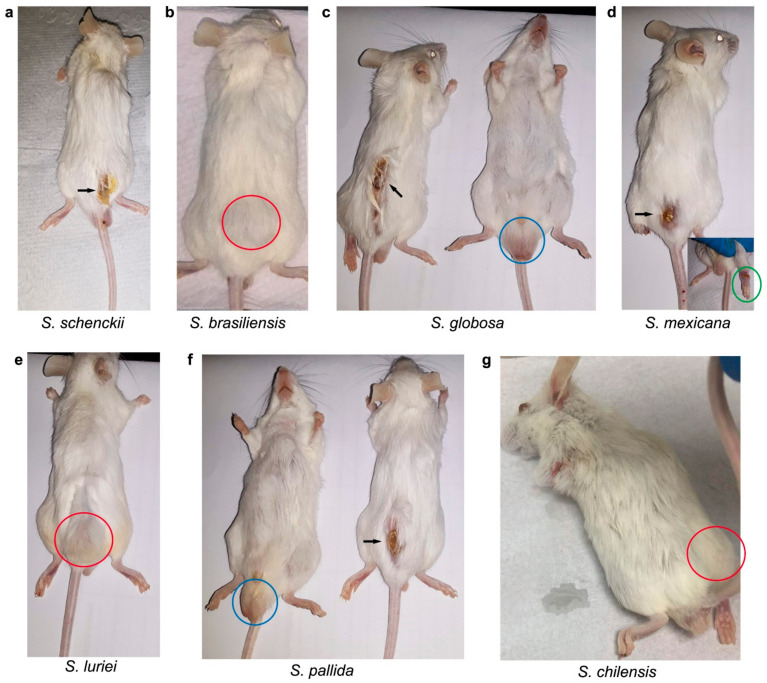
Immunocompetent mice infected with of 3 × 10^6^ conidia of the seven pathogenic *Sporothrix* species, at 35 days after inoculation. The black arrows show ulcerated lesions. The red circles highlight non-ulcerated nodules at the site of inoculation and the blue circles show enlarged testicles. (**a**) ICI/*S. schenckii sensu stricto*; (**b**) ICI/*S. brasiliensis*; (**c**) ICI/*S. globosa*; (**d**) ICI/*S. mexicana* (the green circle highlights the ulcerated lesion in the right paw, characteristic of sporotrichosis); (**e**) ICI/*S. luriei*; (**f**) ICI/*S. pallida* and (**g**) ICI/*S. chilensis*.

At day 21 after infection, ICI mice infected with *S. schenckii sensu stricto*, *S. mexicana*, and *S. pallida* showed edema, flushing, and alopecia at the inoculation site, suggesting a discreet inflammation. No additional clinical signs of sporotrichosis were observed in ICI mice at 21 d.p.i (days post infection). Thirty-five days after inoculation, ICI mice had cutaneous lesions whose severity varied according to the inoculated species. The mice infected with *S. schenckii sensu stricto*, *S. brasiliensis*, *S. globosa*, *S. pallida*, and *S. mexicana* had more extensive ulcerated or nodular lesions, and one mouse infected with *S. mexicana* developed non-ulcerated lesion and ulcerated nodules in the right hind paw (collected for histopathological analysis) (Figure 1). In addition, mice infected with these five species were apathetic, while those infected with *S. luriei* and *S. chilensis* showed no behavioral changes.

**Figure 2:**
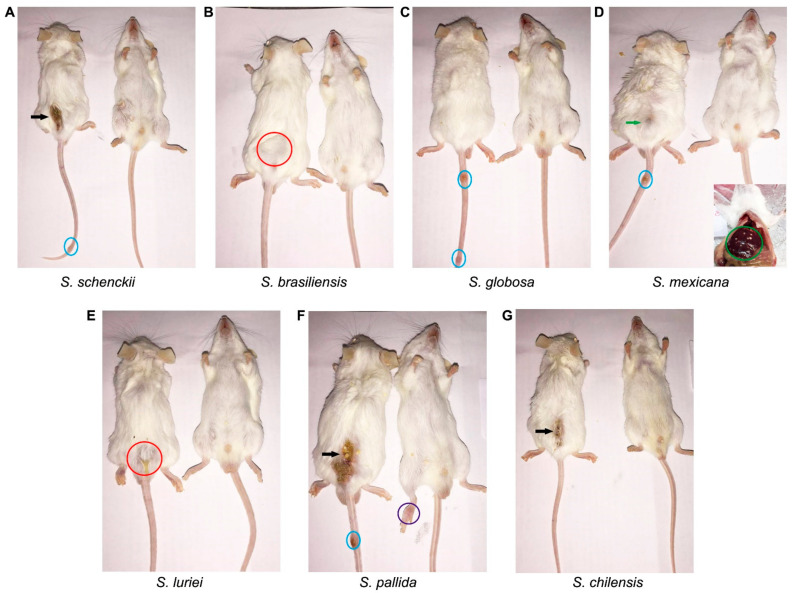
Immunocompetent mice infected with of 3 × 10^6^ conidia of the seven pathogenic *Sporothrix* species, at 49 days after inoculation. The black arrows show ulcerated lesions. The red circles highlight non-ulcerated nodules at the site of inoculation and the blue circles show spread lesions in the tail. (**A**) ICI/*S. schenckii sensu stricto*; (**B**) ICI *S. brasiliensis*; (**C**) ICI/*S. globosa*; (**D**) ICI/*S. mexicana* (the green circle highlights multiple white inflammation foci in the liver and the green arrow points to the beginning of an ulcerated lesion); (**E**) ICI/*S. luriei*; (**F**) ICI/*S. pallida* and (**G**) ICI/*S. chilensis*.

Multifocal lesions and severe splenomegaly were observed in ICI mice infected with *S. schenckii* and *S. mexicana* species 49 days after inoculation, compared with the 35th day. The nodular and/or ulcerated lesions at the point of inoculation spread along the tail of the mice infected with these two species, along with *S. globosa* and *S. pallida*. Immunocompetent mice infected with *S.brasiliensis*, *S. luriei*, and *S. chilensis* showed no lesions (Figure 2).

**Figure 3:**
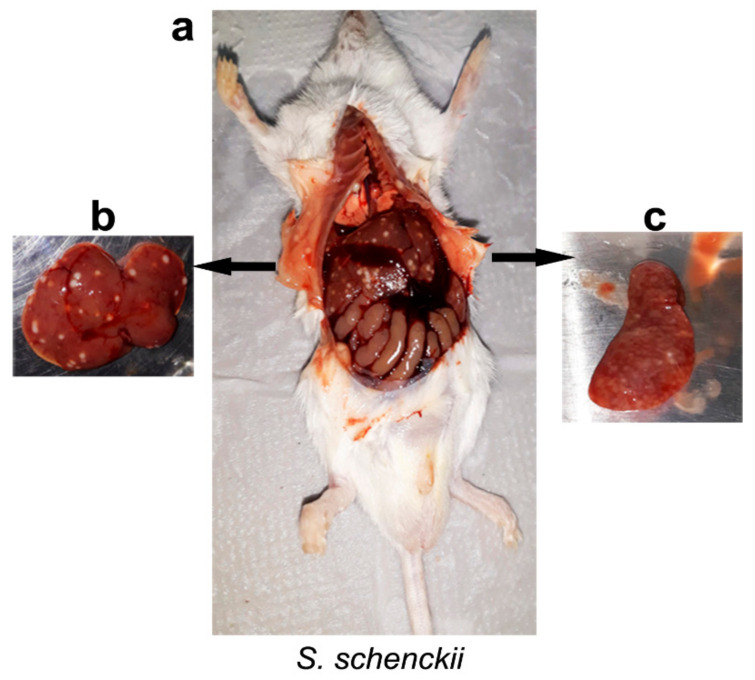
Immunosuppressed mice infected with of 3 × 10^6^ conidia of *S. schenckii* species, at 35 days after inoculation. (**a**) Appearance of thoracic and abdominal cavities with multiple white foci of inflammation; (**b**) Liver; (**c**) Spleen. Cultures of organs, tissue, and purulent material collected from animals of the immunocompetent (ICI) and immunosuppressed (ISI) groups, at all observation times, were positive for *Sporothrix* spp. Control mice from both groups (ICC and ISC) showed no lesions and cultures showed negative.

Unlike those observed in the immunocompetent mice (ICI and ICC), the immunosuppressed groups, infected (ISI) and control (ISC) mice, lost weight, but the *S. schenckii sensu stricto*-infected group (ISI) lost significantly (*p* = 0.0004) more weight compared to the ISC group at 21 and 35 days after inoculation (Table 1). On the 49th day after infection, it was not possible to analyze weight variations due to the 100% mortality of the animals in this group. Mice infected (ISI) with *S. schenckii sensu stricto* and *S. brasiliensis* presented a slight inflammatory reaction at the inoculation point, mild apathy, and hair loss at the first observation point (21 d.p.i.). The group infected with *S. brasiliensis* did not worsen over the observation period, compared to the initial period (21 days), except for a slight spleen atrophy, also observed in the spleen of ISC mice which indicates the intense cellular depletion caused by dexamethasone administration (data not shown). At 35 days after infection, *S. schenckii sensu stricto*-infected mice, despite the action of the immunosuppressive agent, showed marked splenomegaly and multiple white foci of inflammation in the internal organs, especially liver and spleen (Figure 3). On day 49, it was not possible to observe ISI mice infected with *S. schenckii sensu stricto* at this point, due to the 100% mortality of the animals.

### 2.2. Splenic Index, Fungal Burden in the Spleens and Survival Assessment

**Figure 4:**
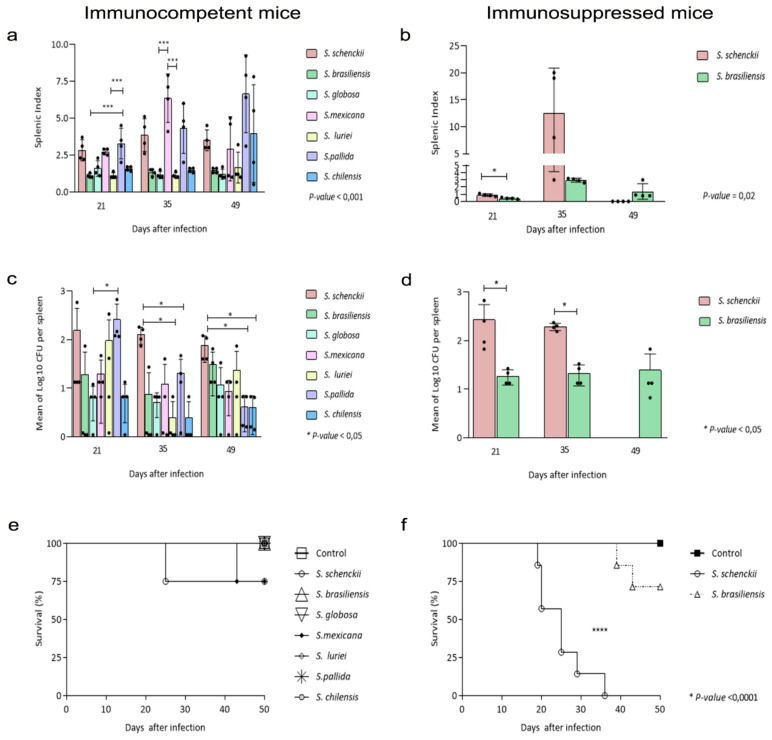
Splenic index value, CFU, and Survival curve of immunocompetent (CI) and immunosuppressed (SI) mice infected with *Sporothrix* spp. and control groups (CC and SC) euthanized 21, 35, and 49 days after inoculation. (**a**,**b**) The control groups (CC/SC) were assigned a splenic index value of 1, therefore, the results described demonstrate how many times the spleen of immunocompetent (**a**) and immunosuppressed (**b**) infected mice is enlarged in relation to its control. (**c**,**d**) Number of *Sporothrix* spp. cells recovered from spleen fragments of immunocompetent (**c**) and immunosuppressed (**d**) mice. The bars represent the experimental groups (CI/SI) and the CFU averages recovered from spleen fragments of four euthanized mice; (**e**,**f**) Survival curve of immunocompetent (**e**) and immunosuppressed (**f**) mice, after inoculation of 3 × 10^6^ conidia of *Sporothrix* species through a subcutaneous route at the base of the tail and a control group similarly inoculated with PBS during the period of 49 days after infection. ANOVA test was used to perform comparisons among CFU and splenic index of the immunocompetent groups. The non-parametric Mann–Whitney u-test was used to analyze the same criteria among immunosuppressed groups. Survival data were analyzed using Kaplan Meier survival plots followed by log rank tests. The *p*-value cutoff for statistical significance was 0.05 and the n value for each group was 4.

In the Figure 4 are demonstrated the data of the splenic index, quantification of colony forming units recovered from the spleens of infected mice and their respective survival curves.

Regarding the mean of splenic index values of (ICI) mice inoculated with the species of *Sporothrix*, at 21 days after inoculation, mice infected with *S. schenckii sensu stricto*, *S. pallida*, and *S. mexicana* had more prominent splenomegaly with statistical significance observed among mice infected with *S. pallida*, *S. luriei*, and *S. brasiliensis* (*p* = 0.001). The splenomegaly in mice infected with *S. mexicana* was clearly marked at the midpoint of infection (35 days). with significant differences among the splenic index of these mice and those infected with *S. globosa* and *S. luriei* (*p* = 0.001). At the last point (49 days), following the decrease in splenic index values of mice infected with *S. mexicana*, it was possible to observe a significant increase of these values in *S. pallida* and *S. chilensis*, however, no statistical differences were observed.

With respect the splenic index of ISI mice infected with *Sporothrix* spp., Figure 4b shows higher splenic index (*p* = 0.02) values from *S. schenckii sensu stricto*-infected mice, 21 days after infection. Statistical differences were not observed on day 35 after inoculation. We again emphasize the absence of comparative data at the last point (49 days), due to the mortality of *S. schenckii sensu stricto*-infected mice. The relative weight of control group spleens (ICC and ISC) was considered one unit.

CFUs were recovered from the splenic tissue culture of ICI and ISI mice at 21, 35, and 49 days after inoculation and at the first point (21 days), we observed that the largest number of fungal cells was recovered from the spleen of *S. pallida* infected-mice, significant in comparison to *S.globosa*-infected mice (*p* = 0.02). On the 35th day after infection, CFU recovered from S*. schenckii sensu stricto*-infected mice was greater than in all others, mainly compared to the mice infected with *S. luriei* and *S. pallida* (*p* = 0.02). On the last point of observation (49 d.p.i.), it is also possible to see the higher number of fungal cells recovered from mice infected with *S. schenckii sensu stricto*, significant compared to *S. pallida* and *S. chilensis*-infected mice (*p* = 0.01). No fungal cells were recovered from control mice spleens.

Similar to that observed in immunocompetent groups, at 21 and 35 days, the number of viable *S. schenckii sensu stricto* cells recovered was higher than that of *S. brasiliensis* in immunosuppressed mice, with a statistical difference between groups (*p* = 0.028). However, it was not possible to make this comparison between species at the last point, due to the mortality of *S. schenckii sensu stricto-*infected mice.

The survival rates of mice inoculated with all species of *Sporothrix* used in the present study are shown in Figure 4e,f. As expected, immunocompetent and immunosuppressed control group (ICC and ISC) mice showed 100% survival until the end of the experiment. Among the ICI mice, *S. schenckii sensu stricto* and *S. mexicana* were able to cause a 25% mortality rate, the former in earlier times of infection, but without statistical differences among groups.

Immunosuppressed mice infected with *S. brasiliensis* had about 60% survival at the end of the observation period, and those infected with *S. schenckii sensu stricto* had total mortality before the 40th day after inoculation. It is noteworthy that the latter group presented strong association between earlier mortality and more pronounced clinical signs of sporotrichosis (*p* < 0.0001).

### 2.3. Histological Studies

**Figure 5:**
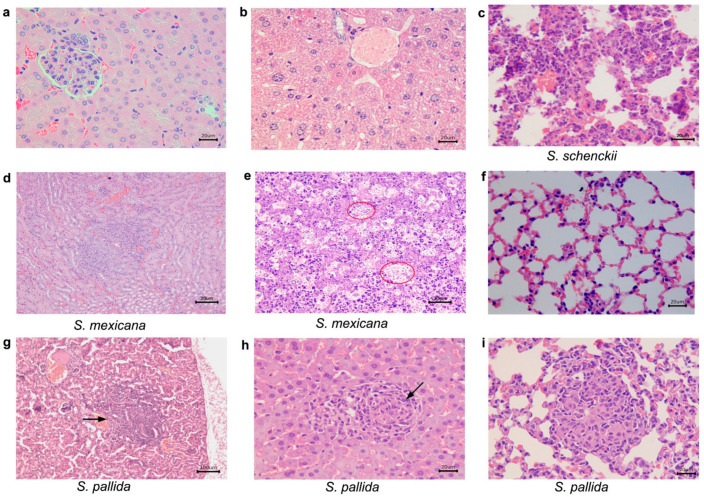
Histological sections of kidney, liver and lungs from immunocompetent mice, inoculated with *Sporothrix schenckii sensu stricto*, *S. mexicana*, and *S. pallida*, observed with hematoxylin-eosin preparations 21 days after inoculation. (**a**,**b**) *S. schenckii sensu stricto*. (**a**) kidney and (**b**) liver without histological alterations; (**c**) *S.* *schenckii sensu stricto*, lung. Pneumonia, pyogranulomatous, and mild; (**d**) *S.* *mexicana*, kidney. Interstitial nephritis, pyogranulomatous, focal, and mild; (**e**) *S.* *mexicana*, liver. Hepatitis, pyogranulomatous, necrotizing. There are abundant yeasts within macrophages amid the inflammatory infiltrate (circles); (**f**) *S. mexicana*, lungs without histological alterations; (**g**) *S. pallida*, kidney. Interstitial nephritis, pyogranulomatous, and moderate in the cortex. (**h**) *S. pallida*, liver. Hepatitis, granulomatous, focal, and moderate. A well-organized granuloma is observed (arrow); (**i**) *S.* *pallida*, lung. Pneumonia, granulomatous, multifocal, and moderate. A well-organized granuloma is observed (arrow).

Details of histopathological analysis of immunocompetent mice infected with *S. schencki sensu strictoi*, *S. mexicana*, and *S. pallida* are described in Table 2. Briefly, in all observation time points (21, 35, and 49 days after infection), immunocompetent mice infected (ICI) with *S. brasiliensis*, *S. globosa*, *S. luriei*, and *S. chilensis*, as well as the control group, (ICC) showed no histological alterations. In contrast, at 21 days after infection, immunocompetent *S. schenckii sensu stricto* infected mice presented lung alterations, while mice infected with *S. mexicana* presented hepatic and renal damage. In mice infected with *S. pallida* it was possible to note lesions in the lungs, liver, and kidney (Figure 5; Table 2).

**Figure 6:**
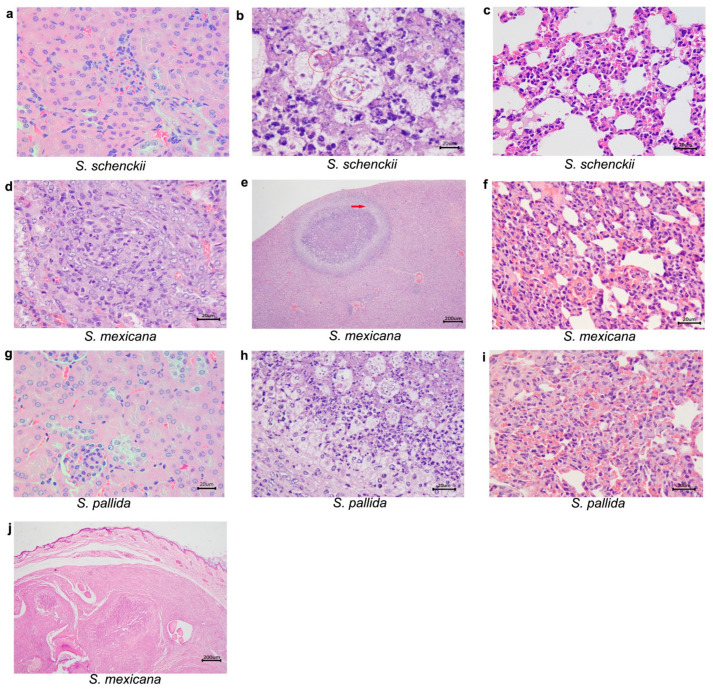
Histological sections of kidney, liver and lungs from immunocompetent mice, inoculated with *Sporothrix schenckii* *sensu stricto*, *S. mexicana*, and *S. pallida*, observed with hematoxylin-eosin preparations 35 days after inoculation. (**a**) *S. schenckii sensu stricto*, kidney without histological alterations; (**b**) *S. schenckii sensu stricto*, liver. Hepatitis, pyogranulomatous, and moderate. Yeasts are observed within macrophages amid the inflammatory infiltrate (circles); (**c**) *S.* *schenckii sensu stricto*, lung. Pneumonia, granulomatous, diffuse, and severe; (**d**) *S.* *mexicana*, kidney. Interstitial nephritis, pyogranulomatous, focal, and discreet in the cortex; (**e**) *S. mexicana*, liver. Hepatitis, pyogranulomatous, necrotizing, and moderate. A well-organized granuloma is observed (arrow). (**f**) *S.* *mexicana*, lung. Pneumonia, pyogranulomatous, diffuse, and moderate; (**g**) *S. pallida*, kidney without histological alterations; (**h**) *S. pallida*, liver. Hepatitis, suppurative infiltrate, multifocal, and mild. (**i**) *S. pallida*, lung. Pneumonia, granulomatous, diffuse, and moderate (**j**) *S. mexicana*, a nodule in the lower right paw. Panniculitis, pyogranulomatous, diffuse, and severe.

Figure 6 shows histopathological alterations in tissues of mice infected at 35 days after inoculation. *S. schenckii sensu stricto*-infected mice presented, in addition to lung damage, liver alterations. At this point, the left hind paw was collected from one mouse infected with *S. mexicana*, containing a nodule for histopathological analysis. It was possible to observe the following histological alterations in the paw tissue: dermatitis, suppurative, diffuse and moderate; panniculitis, pyogranulomatous, diffuse and severe, with multiple well-organized granulomas, with centralizing degenerating neutrophils; extensive areas of fibrosis were also observed in the dermis and subcutaneous tissue; osteomyelitis, pyogranulomatous, diffuse and moderate; and hyaline cartilage degeneration. Additionally, histological alterations were observed in the liver, kidney, and lungs, from those mice infected with *S. mexicana*. Regarding mice infected with *S. pallida*, it was also possible to observe histological changes in the lungs and liver.

**Figure 7:**
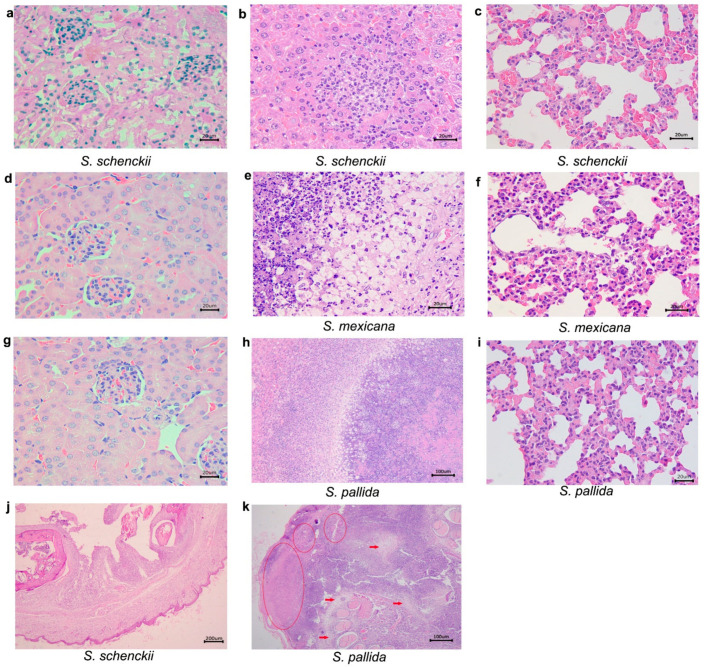
Histological sections of kidney, liver and lungs from immunocompetent mice, inoculated with *Sporothrix schenckii* *sensu stricto*, *S. mexicana*, and *S. pallida*, observed with hematoxylin-eosin preparations 49 days after inoculation. (**a**) *S. schenckii sensu stricto*, kidney without histological alterations; (**b**) *S. schenckii sensu stricto*, liver. Hepatitis, pyogranulomatous, necrotizing, and moderate; (**c**) *S.* *schenckii*, *sensu stricto* lung. Pneumonia, suppurative, diffuse, and mild; (**d**) *S. mexicana*, kidney without histological alterations; (**e**) *S.* *mexicana*, liver. Hepatitis, pygranulomatous, necrotizing, multifocal, and severe; (**f**) *S. mexicana*, lung. Pneumonia, suppurative diffuse, and moderate; (**g**) *S. pallida*, kidney without histological alterations; (**h**) *S. pallida*, liver. Hepatitis, pyogranulomatous, necrotizing, multifocal, and severe; (**i**) *S.pallida*, lung. Pneumonia, suppurative, diffuse, and moderate; (**j**) *S.* *schenckii sensu stricto*, a nodule in the lower right paw. Dermatitis, suppurative, diffuse, and moderate, and panniculitis, pyogranulomatous, diffuse, and severe; (**k**) *S.* *pallida*, tail. Multiple areas of liquefactive necrosis (circles), and fibrosis (arrows) were observed.

It was demonstrated in Figure 7 the histological alterations in organs of infected mice at day 49 after infection. *S. schenckii sensu stricto*-infected mice presented lesions in the lungs and liver. Additionally, one mouse infected with *S. schenckii sensu stricto* presented a nodule in the lower right paw, similar to what was observed in the mice infected with *S. mexicana* at day 35 that was also collected for histopathological analysis. The microscopic examination of the nodule revealed the following histological alterations: dermatitis, pyogranulomatous, diffuse and severe; panniculitis, diffuse and severe pyogranulomatous, with multiple well-organized granulomas, centralizing degenerating neutrophils; degeneration of hyaline cartilage; myositis, pyogranulomatous, diffuse (Figure 7).

The same figure mice infected with *S. mexicana* presented alterations in the lungs and liver, and with respect to the immunocompetent mice infected with *S. pallida*, they also presented alterations in the lungs and liver. The histopathological analysis of the tail showed that the skin had multiple ulcers covered by crust. Dermatitis, panniculitis, myositis and osteomyelitis, and diffuse and severe pyogranulomatous were observed. Cartilage degeneration and bone lysis, as well as multiple areas of liquefactive necrosis and fibrosis were observed.

**Figure 8:**
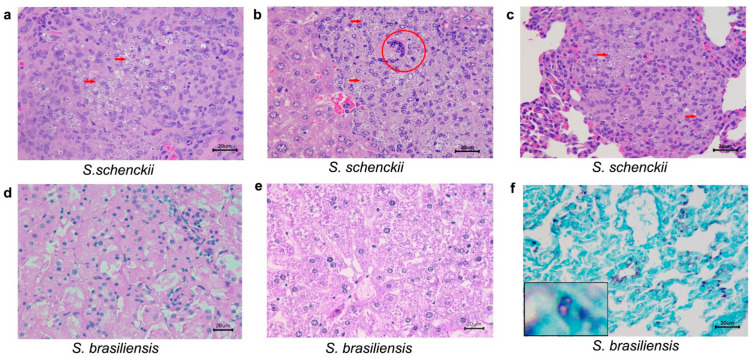
Histological sections of kidneys, liver and lungs from immunosuppressed mice inoculated with *S. schenckii sensu* *stricto* and *S. brasiliensis.* (**a**–**c**) *S. schenckii* at 35 days after infection. Hematoxilin-eosin stain. (**a**) *S. schenckii*, *sensu stricto* kidney. Interstitial nephritis, granulomatous, multifocal, and severe in the cortex and medulla. Multiple yeasts (arrow) were observed within macrophages; (**b**) *S. schenckii sensu stricto*, liver. Hepatitis, pyogranulomatous, necrotizing, multifocal, and severe. Multiple yeasts (arrow) within macrophages and a multinicleated giant cell (MGC–circle) were observed amid the inflammatory infiltrate; (**c**) *S. schenckii sensu stricto*, lung. Pneumonia, pyogranulomatous, and severe. Multiple yeasts (arrow) were observed within macrophages amid the inflammatory infiltrate; (**d**–**f**) *S. brasiliensis* at 35 days after infection. (**d**,**e**) *S. brasiliensis.* Kidney (**d**) and liver (**e**) without histological alterations; (**f**) *S. brasiliensis*; lung stained with Grocott’s methenamine silver (GMS). Black-stained rounded yeasts (inset).

Table 3 shows data concerning histopatological analysis of immunosuppressed mice inoculated with *S. schenckii sensu stricto and S. brasiliensis* (ISI) and the control group (ISC). First, in mice infected (SI) with *S. schenckii sensu stricto*, no alterations were seen 21 days after infection. However, at day 35, all collected organs (lungs, kidney, and liver) presented lesions. Due to total mortality of this group, it was not possible to attain histopathological analysis at 49 days after infection. Regarding immunosuppressed mice infected with *S. brasiliensis*, at 21 and 35 days after infection, no histological alterations were observed. However, at the end of the observation period (49 days), although histological changes were not detected, fungal structures were observed in the GMS staining (Figure 8).

**Figure 9:**
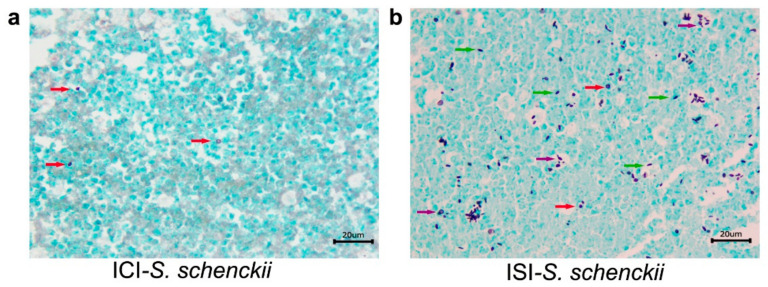
Histological sections of tissues from mice infected with of 3 × 10^6^ conidia of *S. schenckii.* (**a**) Nodule collected of the lower right paw from one immunocompetent mouse, at 35 days after infection; (**b**) Nodule collected of the tail region from one immunosuppressed mouse at the 49 days after infection. Red arrows point black-stained rounded yeasts structures and green arrows show black-stained cigar-shape yeasts. Highlighted by purple arrows, narrow based budding yeasts. GMS.

**Figure 10:**
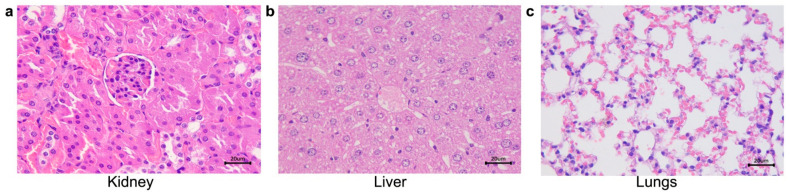
Histological sections of organs from control mice inoculated with PBS, showing no histological alterations. (**a**) Kidney; (**b**) Liver; (**c**) Lungs. HE.

We also demonstrated the differences between number of fungal cells in the nodules collected off the lower right paw from immunocompetent mice, as well as the tail region of immunosuppressed mice infected with *S. schenckii* *sensu stricto*, stained with GMS (Figure 9). In the former, it is possible to see rare, rounded yeast structures in the inflammatory infiltrate, and in the latter, the presence of a large number of fungal cells in the histological section of the tail region. Histological alterations were not seen in organs from mice of the control groups (ICC and ISC) at any of the observed times (Figure 10).

## 3. Discussion

In this study we infected BALB/c mice with one isolate of each of seven species of *Sporothrix* from pathogenic and environmental clades and evaluated clinical and anatomopathological changes in immunocompetent and immunosuppressed murine model. Our research group is a pioneer in studying the infection of mice inoculated simultaneously with isolates of seven clinically important *Sporothrix* species in vivo and exploring the infection with *S. schenckii sensu stricto* and *S. brasiliensis* in a chemically immunosuppressed murine model [24]. Besides that, we have experience in animal models challenged with different fungal species [25,26,27,28,29].

It was possible to observe different biological behaviors among the isolates investigated and establish a ranking of “aggressiveness”, in which the pathogenic *S. schenckii sensu stricto* isolate, followed by *S. mexicana* isolate and, surprisingly, *S. pallida* isolate, the last two, species grouped in the environmental clade, were able to cause more expressive clinical and anatomopathological changes in mice infected with them. It is important to note that *S. schenckii*, and *S. mexicana* have been isolated from patients with some degree of immunosuppression [5,6], but *S. pallida* strain was isolated from soil in Spain [30]. Despite of some experimental studies of virulence concluding that this species has low virulence [31], it has emerged as potentially pathogenic for both humans [7] and animals [21,32], as demonstrated in our study.

Almeida–Paes et al. [33] evaluated phenotypic characteristics associated with virulence of clinical isolates from the *Sporothrix* complex and verified an increased pathogenicity of the *S. brasiliensis* strain in comparison with the *S. schenckii* strain to murine model. The findings of Arrilaga–Moncrief [34], which investigated two isolates pear each of five species of *Sporothrix*, showed that *S. brasiliensis* was the most pathogenic species in mice followed by *S. schenckii* and then *S. globosa*. These authors [34] also found that environmental isolates of *S. mexicana* and *S. albicans* showed low or no virulence in mice. The findings of the present study were different from these authors [33,34], because, as described above, *S. schencki sensu stricto*, *S. mexicana* and *S. pallida* isolates were the most pathogenic in mice. However, clinical isolates of *S. brasiliensis*, *S. globosa*, *S. luriei*, and *S. chilensis* showed no histological alterations. Similar to the present study, Fernandes et al. [31] observed that some isolates of S. *schenckii sensu stricto* were more pathogenic than *S. brasiliensis*.

It is known that the variation in pathogenicity among *Sporothrix* species may be due to several factors as: strains genetic variability, different mice lineages, inoculum concentration and/or to the type of inoculation route (subcutaneous or intravenous) [29]. Our results indicate, in agreement with other authors, that intraspecific genetic differences among the isolates of each species can influence their degree of aggressiveness [25,31]. From our point of view, when we compare our results with authors that studied the same strains used in this paper, it is possible to see similar results to those published by Rodrigues et al. [9] and Arrilaga-Moncrief et al. [34], using, respectively, the same *S. chilensis* and *S. pallida* isolates, used by us.

On the other hand, one of the two *S. brasiliensis* strains investigated by Arrilaga-Moncrief et al. [34], CBS120339, was the same we evaluated and it is from the collection of our institution. These authors showed that it was one of the most virulent species, which was different from our findings. It is interesting to note that the isolate CBS120339 has been preserved for a longer time in the laboratory compared to the isolate used by Arrilaga-Moncrief et al. [34]. It is important to emphasize that preservation methods can cause changes, which may be irreversible, in the biochemical activity, morphological stability and virulence profile of fungal species. It is possible that the preservation method beyond the time to which this strain was kept caused changes in its virulence, not being reversible with our methodology of reactivation of fungi.

When we compare our results with other authors that used different strains can see similarities and differences. It makes us to believe that exist genetic variations between isolates belonging the same species. For example, Brito et al. [28] compared two clinical isolates of *S. schenckii* in murine model, using the same protocol used for us, and showed different virulence profiles between them. Arrilaga-Moncrief et al. [34] showed that *S. globosa* was less virulent than *S. schenckii*, and *S. mexicana* and *S*. *albicans* (current time = *S. pallida*) showed low or no virulence in their animal model. Here we can see similarity of *S. schenckii* virulence results with our data and differences in the results in relation to *S. mexicana* and *S. pallida* data. Oliveira et al. [25] evaluate the virulence of two *S. brasiliensis* isolates from the same patient, with the aim of better understand the differences between them and their relevance to the pathogenesis of the disease and showed that one strain was more aggressive than other in murine model. Just like these authors we used the same lineage of mice, from the same breeding center, under the same age, weight and sex conditions and it is plausible to affirm that the difference in pathological changes observed here is due to diversity of the fungal isolates.

Regarding clinical signs, Cruz–Choappa and colleagues [8], studying mice infected with *S. globosa*, observed that they showed no sporotrichoid lesions and suffering signs, except nodules in the inoculation point, 30 days after infection. In our work, this pattern was observed at day 21, in mice infected with *S. schenckii*, *S. mexicana*, and *S. pallida*, progressively until the end of the experiment, in addition to lesions on the paw and tail, as observed by Nobre et al. [35]. Mice infected with *S. globosa* presented these nodules at day 35, as well as those infected by *S. brasiliensis*, according to that described by Batista-Duharte and collaborators [36]. Della Terra and colleagues [37] used weight loss as a virulence measure because it is correlated with mortality rate, and their results show critical weight loss and mortality of *S. brasiliensis*-infected mice. Our results with all species diverge from these data and resemble those observed by Oliveira and colleagues [27] which described no weight variations in the infected immunocompetent mice.

Arrillaga–Moncrieff and colleagues [34] described that 100% of mice infected with *S. mexicana*, *S. pallida* (formerly *S. albicans*), and *S. globosa* survived until day 40 after infection, and only mice infected with *S. brasiliensis* and *S. schenckii* died over that period. Similar results were found by Fernandes and collaborators [31], showing that mice infected with some *S. brasiliensis* strains died the second week after infection, and *S. schenckii* caused the mortality of the mice in the fourth week. Our surveillance study showed that only immunocompetent mice infected with *S. schenckii* and *S. mexicana* died over the course of the experiment, the former in earlier times. Once again, *S. brasiliensis* clinical isolate was not able to induce mortality as described by some authors [26]. It corroborates the affirmation of Fernandes et al. [31] that there is an intraspecific diversity among strains of *S. brasiliensis* able to influence the pathogenicity of them.

It is possible to assume that the mortality of the immunocompetent mice infected with *S. schenckii* and *S. mexicana*, was caused by hepatitis and/or pneumonia and/or kidney lesions developed by these animals by 21 days after infection. However, the absent or undetectable fungal load in the tissues of mice, inoculated with *S. brasiliensis*, *S. globosa*, *S. luriei*, and *S. chilensis*, suggests low aggressiveness of the isolates evaluated and/or an effective immune response of BALB/c mice against the infection with these isolates. Even the mice that developed the disease, in general, it was possible to note the presence of well-organized granulomas in the tissues. Histopathological lesions in mice infected with species of the clinical clade such as *S. brasiliensis*, *S. schencki sensu stricto* and *S. globosa* were observed by other authors [32,34], but was not reported for the species of environmental clade such as *S. mexicana* and *S. albicans* (sin. *S. pallida*).

Although it was not possible to identify histological changes and / or the presence of the fungus in the organs collected from all the mice, it was feasible to recover, in different amplitudes, fungal cells from the spleens of mice infected with all species. Unlike the findings demonstrated by some authors [8,31,32,37], *S. brasiliensis* did not present an expressive number of viable fungal cells, especially in earlier phases of the infection.

Splenic index values express the splenomegaly attributed to host response to the presence of the fungus in the spleen of infected mice (ICI and ISI). Here, we can see higher values from immunocompetent mice infected with *S. schenckii*, *S. mexicana*, and *S. pallida*, in all the observation points, and at day 49 after infection it is possible to highlight *S. chilensis* as well. Analyzing splenic index of immunosuppressed mice infected with *S. schenckii* and *S. brasiliensis*, the former presents, at day 35 after infection, an expressively higher value. Lewis et al. [38] affirms that, in case of acute or chronic infections, the spleen performs increased immune functions such as clearing antigens and producing antibodies, resulting in splenic hyperplasia, as observed in this work. In addition, it is important to highlight that the glucocorticoids act not only in lymphatic proliferation, but also in the loss of functionality of these cells [24], which can explain the fact that, although there was no atrophy of the spleen, the immunosuppressed mice were still susceptible to the infection.

Immunosuppression is a host condition that can influence the severity of a disease [39], and in our work it was possible to observe that the immunosuppressed groups, especially *S. schenckii*-infected mice, presented such clinical signs as weight loss that could be associated with the 100% mortality of this group after the second time point, as observed by Della Terra and colleagues [37].

Different studies using rodent model demonstrated alterations in the glucose metabolism and suggested that elevations in glucocorticoids (GCs) are tightly linked with diabetes and weight alterations [40,41,42,43,44]. It corroborates our findings that showed that all the animals treated dexamethasone had weight loss, despite increased food intake as observed by Jahng and colleagues [45].

Although some authors have described immunosuppressed experimental models of fungal diseases [46,47], as previously mentioned, data on experimental fungal infection of the *Sporothrix* pathogenic species in immunosuppressed mice are rare. In summary, our study highlights the clinical *S. schenckii* isolate, followed by clinical *S. mexicana*, and environmental *S. pallida* isolates, the last two, species grouped in the environmental clade, as capable of inducing greater anatomopathological changes in mice, which was reflected in the severity of the clinical signs of these animals. It reinforces that not only species-specific but also *Sporothrix* isolate/strain plasticity is one of the factors that can shape the outcome of the infection by this fungus, despite of comparing single strains represents a limitation of this study. Thus, we believe that our data will contribute to research on the use of animal models and, mainly, further studies of virulence profile and taxonomy of species of *Sporothrix* of clinical and environmental origin, necessary to understand the relation between these species and human and animal cases of sporotrichosis, in several regions of world.

## 4. Materials and Methods

### 4.1. Isolates

Seven different *Sporothrix* species were used, all of them previously authenticated by molecular methods. Belonging the pathogenic clade, (a) type strain *S.brasiliensis* IPEC16490/CBS120339 from clinical origin [6], (b) reference strain *S. schenckii* IPEC 27722 isolated from the forearm injury of an immunocompromised patient [6], (c) *S. globosa* IPEC 2713553 isolated from a woman with verrucous lesion on right dorsal hand accompanied of ascending subcutaneous nodules on her arm [48] and (d) *S. luriei* CBS 937.72 isolated from a South African male patient with a tumor on the left frontal region, a fixed type of sporotrichosis [3]. In relation to species belonging the environmental clade, (e) *S. mexicana* MUM 11.02 isolated from a man with multiple polymorphous eruptions and ulcers on both feet [5], (f) *S. pallida* SPA8 an environmental isolate collected from soil in Spain [30], and (g) *S. chilensis* CBS139891 from one onychomycosis clinical case [9]. All species were maintained in the Culture Collection of the Diagnostic Mycology Section of the Evandro Chagas National Institute of Infectious Diseases (INI), Oswaldo Cruz Foundation (Fiocruz), Rio de Janeiro, Brazil. The use of the clinical isolates was approved in accordance with guidelines of the Research Ethics Council of the INI, Fiocruz, under license number 623.950. All the patients or their legal representants informed consent to the use of these samples for research purposes.

### 4.2. Ethics Statement

All experiments involving animal experimentation were conducted according approval by the Animal Use Ethics Committee of the Oswaldo Cruz Institute (CEUA-IOC permit number L-005/2018). This commission was established by IOC Deliberative Council Resolution No. 11/2012 of 5 December 2012, and granted its accreditation on 10 February 2015, process No. 01200.002529/2014-88 and CIAEP No. 01.0234.2014 in compliance with National Council of Animal Experimentation Control (CONCEA).

The animal health and behavior were monitored daily. Since sporotrichosis is a condition that can cause pain from lymphadenopathy, at the time of inoculation and throughout the experimental period, analgesics were administered in the water of the mice. This route of drug administration is effective, non-invasive and does not cause stress in animals. In this way, the non-steroidal anti-inflammatory drug acetaminophen (paracetamol), at a dose of 1 mg/mL, was placed in the water, throughout the 50 days of the experiment.

The humane endpoint was determined by clinical parameters such as behavior (score 0 to 2), presence of injuries (score 0 to 3), clinical signs of discomfort or pain (score 0 to 3) and food intake (score 0 to 5). Mice that presented a sum of parameter scores equal to or greater than 5 were euthanized.

### 4.3. Mice Experimental Inoculation

Four hundred and seventeen BALB/c male mice weighing approximately 25 g, aged 6–8 weeks, from Science and Technology in Biomodels Institute (ICTB/Fiocruz) were used in this study. Twenty-one of these mice were used for fungal reactivation (3 for each isolate) and 396 for clinical and anatomopathological assays, divided into two major groups: immunocompetent, which comprised of 144 mice subdivided into 8 groups and inoculated with each one of the seven *Sporothrix* isolates (ICI) or PBS (ICC) and 54 immunosuppressed, similarly subdivided into 3 groups and inoculated with *S. schenckii*, *S. brasiliensis* (ISI), or PBS (ISC). For each group, 6 mice were used to perform survival curve and 12 mice were used for other evaluations. All mice were maintained in a breeding room kept at room temperature, 23–25 °C, humidity, 30–70%, light/dark cycle, 12 h, and received water and food *ad libitum*. We selected the sample size in order to meet the ethical principles of animal experimentation, without, however, impairing the statistical analysis of the study. We choose a mice lineage inbred with less than 1% of genetic variability [48,49] to perform all the experiments twice, independently, to guarantee the reproducibility of the study and the reliability of the data.

### 4.4. Immunosuppressive Treatments

We also choose to use immunosuppressed mice model to mimic the conditions of the patients affected with the more severe forms of the sporotrichosis. So, to perform immunosuppression mice received 1 mg/kg of dexamethasone (Hypofarma, Ribeirão das Neves, Brazil) administered ad libitum in the drinking water for 3 days before fungal inoculation and during all the experiments [27]. Tetracycline (1000 mg/L, Teuto, São Paulo, Brazil) was also added to the drinking water in parallel in order to prevent bacterial infections. We chose this route of administration of the drugs (in the water) to minimize the stress caused by handling the animals.

### 4.5. Fungal Reactivation

Fungal reactivation was performed to check the viability, stability and to restore the pathogenicity of the strains stored for long periods in culture collections. *Sporothrix* isolates were cultured in Sabouraud broth (DifcoTM Becton, Dickinson and Company/Sparks, MD 21152, USA) at 25 °C with shaking at 100 oscillations/min. After 11 days, the fungal growth was filtered through sterile gauze and conidia were pelleted by centrifugation at 3000 *g* for 5 min. After 3 washes, resuspended in 1 mL of PBS (50 mM phosphate-buffered saline at pH 7.2) and counted with a Neubauer chamber, and their viability determined by colony forming unit (CFU) protocol [49]. After that, 21 mice (3 for each isolate) were inoculated intraperitoneally with 3×10^6^ conidia of each isolate in 0.02 mL of sterile PBS, and after 20 days subjected to euthanasia by anesthetics overdose (ketamine—90 mg/kg and xylazine—10 mg/kg). The isolates were recovered through culture of the spleens on Mycosel agar (Becton, Dickinson and Company, Sparks, MD, USA) at 37 °C.

We choose to use conidia to perform experimental mice infections because *Sporothrix* is a dimorphic fungus and in its filamentous phase the fungus is able to infect the mammalian host through traumatic implantation of conidia in the subcutaneous tissue and only after infection, it converts to the yeast-like phase.

In agreement of the statement of the ethics committee, were used the minimum number of animals per species, to ensure the recovery of fungal cells.

### 4.6. Fungal Inoculation

To perform the experiments, conidia were obtained in the same way as described for fungal reactivation, and mice were inoculated through a subcutaneous route at the base of the tail, with 3 × 10^6^/conidia, according to Corrêa–Moreira [24], with more than 87% viability. The control group was similarly injected with PBS.

### 4.7. Euthanasia, Necropsy, CFU Determination, Splenic Index, and Survival Assessment

Clinical manifestations as external lesions, apathy, and alopecia were observed. At 21, 35, and 49 days after fungal inoculation, four mice from each group were weighed, euthanized by anesthetics overdose using ketamine (90 mg/kg) and xylazine (10 mg/kg). After a macroscopic examination of the internal organs, the spleen, lungs, kidneys, and liver were removed aseptically. The spleens were weighed and homogenized in a sterile, complete RPMI-1640 medium containing L-glutamine (Sigma Chemical, St Louis, MO, United States) to determine the number of CFU. The suspension was adjusted to 2 mg of tissue per ml and samples of 150 µL of each homogenate were transferred to Petri dishes with Mycosel agar (Becton Dickinson and Company, Sparks, MD, United States), incubated at 37 °C for 15 days, for fungal re-isolation and quantification of the colony forming unit. The spleen and body weight ratios of each infected mouse and of control mice were also determined. For calculation of the splenic index, the ratios of relative weight of spleens from infected mice were expressed as units in relation to the control. The mean value for the relative weight of spleens in each control group of mice was considered to be equal to one unit [50]. The survival assessment of the immunocompetent and immunosuppressed mice was observed for 50 days following inoculation. Mortality in each group was noted daily.

### 4.8. Histopathology

The livers, lungs, kidneys, and hearts collected in the necropsy were immediately fixed in 10% buffered formalin, embedded in paraffin, sectioned and placed on slides, and stained with hematoxylin-eosin (HE) and Grocott’s methenamine silver (GMS). According to the cell types in the inflammatory infiltrate, they were classified as granulomatous (predominance of cells of the monocyte-macrophage system such as activated macrophages, epithelioid macrophages, or multinucleate giant cells); pyogranulomatous (predominance of cells of the monocyte-macrophage system and large number of neutrophils); and as non-granulomatous (predominance of other cell types). When there were neutrophils, the non-granulomatous infiltrate was classified as suppurative, and as lymphoplasmacytic when composed primarily of lymphocytes and plasma cells without neutrophils. About the distribution, the inflammatory infiltrate was classified as: focal (1 inflammatory focus), multifocal (more than 1 inflammatory focus), and diffuse (inflammatory cells evenly distributed in the tissue section). The granuloma was classified as well organized (nodular) or poorly organized (diffuse). The intensity of inflammatory infiltration was classified as absent, mild (mild and dispersed foci), or moderate to intense (dense and diffuse cellular infiltrate). The presence of *Sporothrix* spp. yeasts in the tissues was confirmed by GMS-stain.

### 4.9. Statistical Analysis

Kruskal–Wallis one-way ANOVA followed by Dunn’s multiple comparison post hoc nonparametric tests were used to pinpoint which specific immunocompetent group means were significantly different from the others within different times after infection regarding its CFU, weight loss, and splenic index. Mann–Whitney U-test was used to test significant means difference between immunosuppressed groups within the same times after infection for the same dependent variables. Kaplan–Meier curves were used to describe the survival of mice, and Log-rank tests were used to compare estimates of the hazard functions of infected groups against control at 50 days after infection. Two-tailed level of significance, alpha = 0.05, were adopted for all tests (n-samples = 4). Statistical analyses were conducted in Prism GraphPad for Windows version 8 (GraphPad Software, San Diego, CA, USA).

## Figures and Tables

**Table 1:** Weight variation of immunocompetent and immunosuppressed mice infected with *Sporothrix* species and control groups.

Species	Days after Inoculation	Mean Weigh of Mice (g) ± SD	
		Immunocompetent	Immunosuppressed
*S. schenckii sensu stricto*	*21*	26.66 ± 1.35	22.86 ** ± 1.49
	35	28.44 ± 1.78	19.96 ** ± 2.76
	49	27.37 ± 2.96	Ø
*S. brasiliensis*	21	30.82 ± 3.28	23.83 ± 0.73
	35	31.22 ± 1.56	25.92 ± 1.65
	49	31.26 ± 0.65	23.81 ± 1.44
*S. globosa*	21	26.65 ± 1.86	
	35	30.67 ± 1.35	N.I.
	49	29.66 ± 1.01	
*S. mexicana*	21	26.57 ± 1.85	
	35	26.51 ± 3.12	N.I.
	49	29.97 ± 1.85	
*S. luriei*	21	27.61 ± 1.21	
	35	30.31 ± 0.71	N.I.
	49	30.94 ± 0.64	
*S. pallida*	21	27.23 ± 0.52	
	35	29.52 ± 2.20	N.I.
	49	26.81 ± 2.37	
*S. chilensis*	21	30.15 ± 1.65	
	35	30.45 ± 2.01	N.I.
	49	28.53 ± 0.58	
CONTROL	21	28.96 ± 0.42	24.30 ± 1.42
	35	30.44 ± 20.1	23.15 ± 1.06
	49	29.30 ± 2.60	23.58 ± 0.97

SD: Standart deviation. ** Statistical difference between immunosuppressed infected mice (SI) and control group (SC). (*p*-value = 0.0004). Ø All mice had died at this time point. N.I.: Mice non-inoculated with this species.

**Table 2:** Histological alterations in the liver, lungs, and kidneys of immunocompetent mice inoculated with the *Sporothrix* spp. observed with hematoxylin-eosin and Grocott’s methenamine silver (GMS) preparations at 21, 35, and 49 days after inoculation.

Days after Inoculation	*Sporothrix* Species	Organs		
		Kidney	Liver	Lungs
**21**	***S. schenckii sensu stricto***	ABSENT	ABSENT	Pneumonia, pyogranulomatous, multifocal and mild. Poorly organized granuloma. GMS: rounded yeasts amid the inflammatory infiltrate.
	***S.mexicana***	Interstitial nephritis, pyogranulomatous, focal and mild. Poorly organized granuloma. GMS: remaining fungal cell wall amid the inflammatory infiltrate.	Hepatitis, pyogranulomatous, necrotizing, multifocal and moderate, with areas of fibrosis. Poorly organized granuloma. GMS: Rounded yeasts within the granulomas.	ABSENT
	***S. pallida***	Interstitial nephritis, pyogranulomatous, multifocal and moderate in the cortex. Poorly organized granuloma. GMS: rare rounded yeasts amid the inflammatory infiltrate.	Hepatitis, granulomatous, focal and moderate. Well organized granuloma. GMS: Rounded and cigar shaped yeasts, amid the inflammatory infiltrate.	Pneumonia, granulomatous multifocal and moderate to severe. Well organized granuloma. GMS: rounded yeasts, amid the inflammatory infiltrate.
**35**	***S. schenckii sensu stricto***	ABSENT	Hepatitis, pyogranulomatous, multifocal and moderate. Poorly organized granuloma. GMS: fungal structures were not detected.	Pneumonia, granulomatous, diffuse and moderate to severe. Poorly organized granuloma. GMS: fungal structures were not detected.
	***S. mexicana***	Interstitial nephritis, pyogranulomatous, focal and discreet in the cortex. Poorly organized granuloma. GMS: fungal structures were not detected.	Hepatitis, pyogranulomatous, necrotizing, multifocal and moderate. Multiple fibrosis areas. Well organized granuloma. GMS: rounded with a narrow-based budding or cigar-shaped yeasts within granulomas.	Pneumonia, pyogranulomatous, diffuse and moderate to severe. Poorly organized granuloma. GMS: rare rounded yeast forms amid the inflammatory infiltrate.
	***S. pallida***	ABSENT	Hepatitis, suppurative or lymphoplasmacytic infiltrate, multifocal and mild. Poorly organized granuloma. GMS: Rounded yeasts within the granulomas.	Pneumonia, granulomatous, diffuse and moderate to severe. Poorly organized granuloma. GMS: fungal structures were not detected.
**49**	***S. schenckii sensu stricto***	ABSENT	Hepatitis, pyogranulomatous, necrotizing, multifocal and moderate to severe. Well organized granulomas. GMS: rare round yeast forms within the granulomas.	Pneumonia, suppurative, diffuse and mild. GMS: fungal structures were not detected.
	***S. mexicana***	ABSENT	Hepatitis, pyogranulomatous, multifocal and moderate. Multifocal areas of fibrosis. Poorly organized granuloma. GMS: rounded and cigar shaped yeasts, amid the inflammatory infiltrate.	Pneumonia, suppurative, diffuse and moderate. Poorly organized granuloma. GMS: fungal structures were not detected.
	***S. pallida***	ABSENT	Hepatitis, pyogranulomatous, multifocal and moderate to severe. Poorly organized granuloma. GMS: rounded or cigar shaped yeasts within the granuloma amid necrosis areas.	Pneumonia, suppurative, diffuse and moderate to severe. Poorly organized granuloma. GMS: fungal structures were not detected.
	**CONTROL**	ABSENT	ABSENT	ABSENT

ABSENT: Tissue alterations and yeasts of *Sporothrix* spp. were not detected by hematoxylin-eosin and Grocott’s methenamine silver (GMS) preparations.

**Table 3:** Histological alterations in the liver, lungs, and kidneys tissues of immunosuppressed mice inoculated with the *S. schenckii sensu stricto* and *S. brasiliensis*, observed with hematoxylin-eosin and Grocott’s methenamine silver (GMS) preparations at different time points.

Days after Inoculation	*Sporothrix* Species		Organs		
			Kidney	Liver	Lungs
**21**	***S. schenckii sensu stricto***	ABSENT		ABSENT	ABSENT
	***S. brasiliensis***	ABSENT		ABSENT	ABSENT
**35**	***S. schenckii sensu stricto***	Interstitial nephritis, granulomatous, multifocal and severe in the capsule, cortex, medulla and pelvis. Multifocal areas of fibrosis. Poorly organized granuloma. GMS: abundant rounded yeasts with a narrow-based budding or cigar-shaped in in the capsule, cortical, medullary region and renal pelvis.		Hepatitis, pyogranulomatous, necrotizing, multifocal and moderate to severe. Well-organized granulomas. GMS: abundant rounded yeasts with a narrow-based budding or cigar-shaped, amid the inflammatory infiltrate	Pneumonia, pyogranulomatous, multifocal and moderate to severe. Abundant yeast-like structures amid the inflammatory infiltrate. GMS: abundant rounded yeasts with a narrow-based budding or cigar-shaped, amid the inflammatory infiltrate
	***S. brasiliensis***	ABSENT		ABSENT	ABSENT
**49**	***S. schenckii sensu stricto***	All animals had died by this point in the study, and histopathological analysis was not possible.			
	***S. brasiliensis***	ABSENT		ABSENT	Tissue alterations were not detected by HE staining. GMS: rounded yeasts with a narrow-based budding or cigar-shaped, amid the inflammatory infiltrate
	**CONTROL** (of all species at different times)	ABSENT		ABSENT	ABSENT

ABSENT: Tissue alterations and yeasts of *Sporothrix* spp. were not detected by hematoxylin-eosin and Grocott’s methenamine silver (GMS) preparations.

## Data Availability

Data sharing is not applicable to this article.
